# Tau pathology in neurodegenerative disease: disease mechanisms and therapeutic avenues

**DOI:** 10.1172/JCI168553

**Published:** 2023-06-15

**Authors:** Niyatee Samudra, Courtney Lane-Donovan, Lawren VandeVrede, Adam L. Boxer

**Affiliations:** Memory and Aging Center, Department of Neurology, and Weill Institute for Neurosciences, UCSF, San Francisco, California, USA.

## Abstract

Tauopathies are disorders associated with tau protein dysfunction and insoluble tau accumulation in the brain at autopsy. Multiple lines of evidence from human disease, as well as nonclinical translational models, suggest that tau has a central pathologic role in these disorders, historically thought to be primarily related to tau gain of toxic function. However, a number of tau-targeting therapies with various mechanisms of action have shown little promise in clinical trials in different tauopathies. We review what is known about tau biology, genetics, and therapeutic mechanisms that have been tested in clinical trials to date. We discuss possible reasons for failures of these therapies, such as use of imperfect nonclinical models that do not predict human effects for drug development; heterogeneity of human tau pathologies which may lead to variable responses to therapy; and ineffective therapeutic mechanisms, such as targeting of the wrong tau species or protein epitope. Innovative approaches to human clinical trials can help address some of the difficulties that have plagued our field’s development of tau-targeting therapies thus far. Despite limited clinical success to date, as we continue to refine our understanding of tau’s pathogenic mechanism(s) in different neurodegenerative diseases, we remain optimistic that tau-targeting therapies will eventually play a central role in the treatment of tauopathies.

## Introduction

Tauopathies are neurodegenerative diseases defined by the accumulation of misfolded, insoluble tau protein aggregates in neuronal and/or glial inclusions detectable in the brain at autopsy (1). These disorders are associated with diverse cognitive, motor, and neuropsychiatric abnormalities (2). Since tau pathologic burden strongly correlates with the severity of neurodegeneration as well as clinical phenomenology, tau has been the focus of therapeutic development, with multiple tau-directed therapeutics evaluated in clinical trials of Alzheimer’s disease and other tauopathies (3, 4).

Tau protein has a physiologic role as a soluble cytoplasmic protein interacting with microtubules, primarily through a microtubule-binding region (MTBR), to stabilize cytoskeleton and regulate axonal transport. It also affects a diverse array of other cellular processes, including synaptic function, gene expression, and energy metabolism (5). It is ubiquitously expressed in neurons (6–9). Tau’s neurodegenerative role rests on multiple lines of evidence. These include genetic and autopsy data from human tauopathies, as well as nonclinical models of disease, such as induced pluripotent stem cell models and transgenic rodents that express mutant forms of tau associated with autosomal dominant frontotemporal dementia (FTD) (10, 11). Given that tau loss of function in animal models does not replicate human clinical phenotypes, and genome-wide screens (GWAS) for disease-associated mutations only identify gain-of-function mutations in the tau-encoding gene, *MAPT*, toxic gain of function has been historically suggested as the cause of tauopathies (12). Abnormal tau protein folding has been thought to lead to cytotoxic tau aggregation, accumulation of insoluble tau deposits, and subsequent neuronal loss that correlates with the clinical features of tauopathies during life in clinical-autopsy and clinical–tau PET studies (13, 14).

Here, we review normal and abnormal tau biology, tau genetics, nonclinical models and their relationship to human disease, and hypotheses regarding proposed roles of tau in neurodegeneration. Despite strong evidence for a central pathologic role of tau in neurodegenerative tauopathies, recent human clinical trials of experimental tau-targeting therapies have failed to demonstrate clinical benefit, including drugs purported to interfere with pathologic aggregation, processing, and accumulation of tau (15, 16).

Despite a variety of potential explanations for the negative clinical trials, the lack of efficacy of tau therapies tested to date has raised important questions regarding what is truly understood about tau and its suitability as a drug target in human neurodegenerative disease. Though tau therapeutics have improved pathology in nonclinical models of tauopathy, do the recent negative human clinical trial results reflect flawed nonclinical models that inadequately model human disease? Are the models potentially predictive of therapeutic effects in human disease, but not the disease(s) in which the therapies were tested? Or is the lack of human efficacy explained by problems related to drug development, such as the wrong therapeutic target, inadequate dose, or lack of target engagement? Given these challenges, is it time to rebalance the approach to development of tau therapeutics using nonclinical models and early-stage human clinical trials?

## Normal and abnormal tau biology

Six tau protein isoforms are encoded from the *MAPT* gene by alternative splicing of exons 2, 3, and 10 (17). In particular, exon 10 alternative splicing can generate isoforms with three or four MTBR repeats (3R or 4R tau) (18). In the normal human adult brain, there are approximately equal concentrations of 3R and 4R tau, and changes in this ratio are associated with several neurodegenerative tauopathies, most commonly a relative overexpression of 4R tau (19).

Tau is one of several neuronal proteins responsible for promoting cytoskeletal microtubule assembly and stability. It may also play other cellular roles through its ability to bind to nucleic acids, and its localization to the synapse and mitochondrial compartments (5). Normally, tau is soluble and natively unfolded, whereas it becomes insoluble when hyperphosphorylated, shifting toward polymerization owing to an increase in β-sheet structures, which is also seen in other protein deposition disorders (20). Abnormal hyperphosphorylation and lack of tau clearance in the disease state is associated with diverse intraneuronal and glial inclusions (21). Decreased reversibility of hyperphosphorylation may contribute to pathogenesis in some tauopathies (22). There are many other posttranslational modifications of tau, including O-GlcNAcylation, acetylation, and glycosylation, that may influence the function and pathology of tau (9, 23).

## Classification of tauopathies and conceptualization of tau dysfunction

Tauopathies are often classified based on the primary tau protein isoform deposited in the brain, including 3R tauopathies, exemplified by Pick’s disease (PiD); 4R tauopathies, such as progressive supranuclear palsy (PSP), corticobasal degeneration (CBD), argyrophilic grain disease (AGD), and globular glial tauopathy (GGT); and combined 3R/4R tauopathies, such as Alzheimer’s disease (AD), chronic traumatic encephalopathy (CTE), and primary age-related tauopathy (PART).

**Figure 1:**
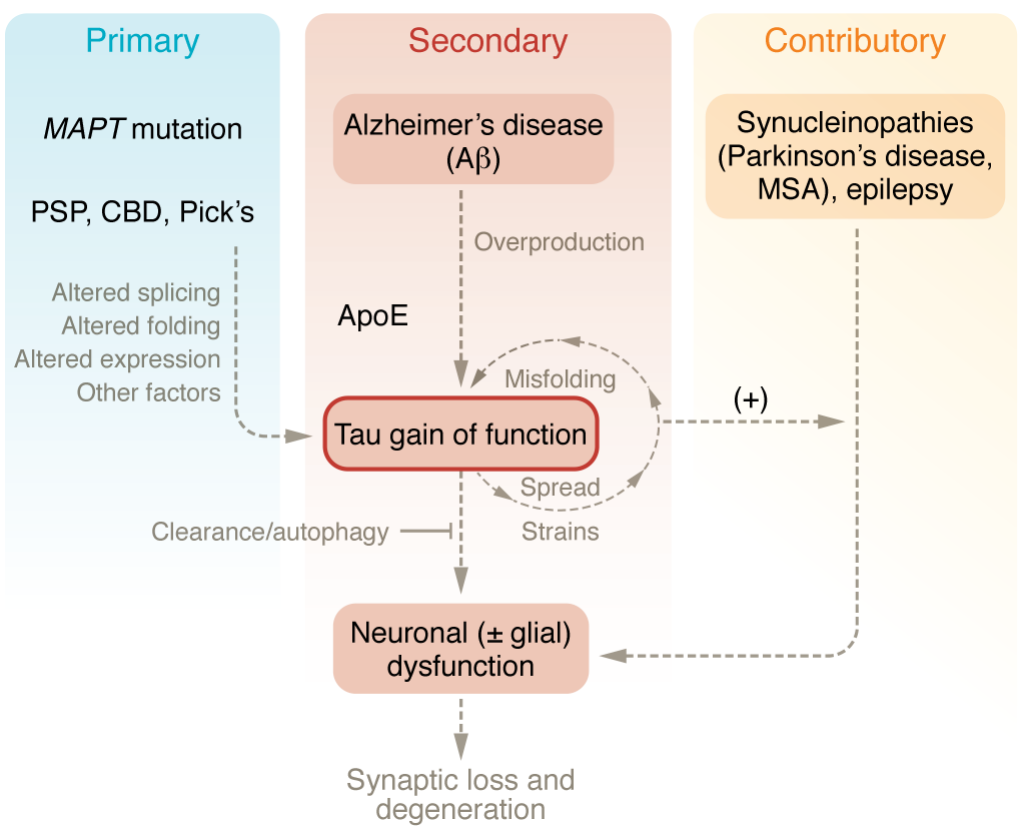
The hypothesized role of tau in degeneration in various tauopathies (primary, secondary, and contributing). In particular, gain of function may lead to misfolding and spread in secondary tauopathies. A similar process may contribute in other disorders (synucleinopathies, epilepsy) that are not classically considered tauopathies.

Tauopathies can also be classified based on whether tau is the only aggregated protein found in the brain at autopsy or whether other proteins or pathogenic events are believed to initiate tau pathology. See Figure 1 for a summary. More than 20 different tauopathies have been identified, considered “primary,” in which tau is the only pathogenic protein found at autopsy, or “secondary,” in which tau pathology may accumulate due to the presence of another pathology (24).

Many data from cell culture and animal models, as well as human neuropathologic correlations, suggest that tau is likely to be a key pathogenic driver in most tauopathies. An alternate, less likely hypothesis is that tau pathology is a permissive factor or an epiphenomenon that correlates with disease pathophysiology (25). Tauopathies where tau abnormalities definitely cause disease are autosomal dominant *MAPT* mutations, which lead to hereditary forms of frontotemporal lobar degeneration. Different *MAPT* mutations are associated with specific clinical phenotypes and biomarker profiles (26). Intron 10 (IVS10) and other *MAPT* mutations that increase 4R tau production often lead to movement disorder phenotypes similar to sporadic PSP or CBD (27). The strongest genetic risk factors for these sporadic primary tauopathies are in and around the *MAPT* gene, including the H1c subhaplotype, which is believed to increase *MAPT* mRNA expression (28). These human genetic data strongly support a central role for tau protein pathogenesis in CBD and PSP. Separately, GWAS evidence suggests that tau may play a role in the pathogenesis of synucleinopathies, such as Parkinson’s disease and multisystem atrophy, as well as certain forms of epilepsy, such as Dravet’s syndrome (29).

The trans-synaptic spread (“prion”) hypothesis of tau spread has garnered recent interest. This hypothesis is supported by the predictable progression intracerebrally of tau protein in various diseases, including AD, correlating with clinical symptoms (30). In AD, the spread of tau neurofibrillary tangles (NFTs) from entorhinal cortex to hippocampus to cortical regions prior to and in tandem with the development of clinical symptoms suggests tau’s causal role (31). In animal and cell culture models, tau spreads in a prion-like manner, potentially explaining the stereotypical pattern of progression of tau accumulation in neurodegenerative diseases like AD (31–33). Seeding-based mouse models expressing human *MAPT* gene (wild type or mutant) have demonstrated conversion of tau monomers to oligomers, and then to insoluble fibrils (34). In these seeding paradigms, mice are injected with lysates from human disease brain, transgenic mouse brain, or in vitro tau aggregates. The seeding can induce tau aggregation and pathology, which can be accelerated by amyloid pathology or age (35, 36). Pattern of distribution and affected cell type can be distinct between each tau strain, often mirroring findings of the initial disease (e.g., oligodendrocyte tau pathology in CBD mice) (37).

It is more difficult to connect tau burden with clinical presentation in “incidental” tauopathies, which are often subclinical in nature, noted as co-pathologies or contributing pathologies in brain autopsies, with phosphorylated tau (p-tau) aggregates, and also termed age-related tauopathies (25). They include pathologies akin to primary age-related tauopathy (PART), aging-related astrogliopathy (ARTAG), and argyrophilic grain disease (AGD). For example, AGD is often comorbid with AD, and has been associated with a prolonged period of amnestic mild cognitive impairment. However, in a significant proportion of cases it may be asymptomatic (38). The existence of these disorders challenges the dogma that NFTs are always necessarily pathogenic, rather than reactive or protective, as neurons with NFTs can survive for decades (39, 40).

## Alternative conceptualizations of tau pathogenicity

Though toxic gain of function has been hypothesized to cause tauopathies, loss of tau physiological function could also contribute (12). Tau protein interacts with more than a hundred targets, including presynaptic, postsynaptic, and mitochondrial proteins (5, 41). Depletion of tau in cells with drug-induced DNA damage increases cell senescence (42). Further, missense mutations in the *MAPT* gene reduce tau’s ability to bind microtubules and promote microtubule assembly, causing an FTD with Parkinsonism phenotype (43).

Other possible mechanisms of pathogenicity relate to downstream effects of tau dysfunction. One potential unifying hypothesis is that age- and/or neurodegeneration-related loss of protein homeostasis leads to an inability to clear soluble tau species that may be pathogenic (44). There is evidence that tau acetylation leads to failed tau clearance by chaperone-mediated autophagy (45, 46). Nucleocytoplasmic and mitochondrial transport may also be impaired by AD-related tau (47, 48).

Neuroinflammation related to tauopathy may also be an important mechanism leading to the development or progression of neurodegenerative disease (49, 50). Tau transgenic mice demonstrate colocalization of tau oligomers with astrocytes, microglia, and inflammatory cytokines (51). Moreover, it was recently shown that tauopathy mouse models have increased parenchymal cytotoxic T cells and microglia, and that depletion of either cell population prevents tau-mediated brain atrophy (50). Autophagy, mitophagy (the specific or selective removal of mitochondria), and neuroinflammation could have a synergistic effect in the development of tauopathy, particularly in AD (52).

Other possible routes of pathogenicity include the interaction of tau with other proteins involved in neurodegenerative disease. Amyloid-β (Aβ) and tau in AD have a pathogenic interaction in human disease (53). In an AD mouse model expressing both human pathologies, tau and Aβ had opposite effects on cortical hyperactivity, and tau gene suppression was ineffective in rescuing neuronal impairments, suggesting a complex interaction (54). Phase III trials in AD have suggested some efficacy of the Aβ-targeting antibodies lecanemab and aducanumab in slowing rates of cognitive decline. Preliminary phase II trials with donanemab also demonstrate lowering of plasma p-tau, suggesting a downstream effect of these agents on AD tau pathology (55–57). Parallels between changes in plasma p-tau species and glial fibrillary acidic protein (GFAP) species in recent anti-amyloid antibody trials (phase II in donanemab and phase III in lecanemab) raise the possibility that astroglial activation may mediate the interaction between Aβ plaques and soluble p-tau accumulation in AD. Further evidence from a human presenilin-1 (*PSEN1*) mutation carrier with a protective apolipoprotein E (apoE) mutation who had reduced tau accumulation and preserved cognition also implicates apoE in this process (58).

Tau, α-synuclein, and TAR DNA-binding protein 43 (TDP-43) appear to have synergistic neurotoxic effects, based on their colocalization in humans at autopsy and in vivo model data (59, 60). Based on spectroscopic analysis, there may be synergistic aggregation between tau and α-synuclein molecules that contributes to neural cytotoxicity (61). Co-pathology of various proteinopathies is very common in neurodegenerative disease and increases with age. This has therapeutic implications in tauopathies since the presence of co-pathologies could mask tau-specific therapeutic effects (62).

## Tau genetics

Mutations in *MAPT* are the cause of autosomal dominant forms of frontotemporal lobar degeneration (FTLD) that present most commonly with behavioral variant FTD, but sometimes with movement disorders. Other mutations, including R406W and V337M, produce mixed 3R/4R tau pathology similar to AD, presenting with an amnestic AD-like syndrome and tau that binds AD tau PET tracers. Overall, nearly 60 mutations in *MAPT* have been identified as pathogenic (63, 64).

A chromosomal inversion in the *MAPT* region defines two major tau haplotypes, H1 and H2. Various reports have mentioned different possible effects of H1 and H2 haplotypes on age of onset of or risk for different neurodegenerative diseases, either alone or in combination with other genes. For example, the combination of H1 haplotype and apolipoprotein E (*APOE*) ε4 allele may increase risk of earlier-onset FTD (65). Many patients with PSP carry the H1 haplotype (66). A subhaplotype of H1, H1c, is linked to PSP and CBD (67, 68). It is also possible that the H2 haplotype may be protective against PSP and CBD, although the mechanisms are not well defined (69, 70). GWAS have also identified shared risk between CBD and PSP at different gene loci that do not involve the *MAPT* gene, including *MOBP*, *CXCR4*, *GLDC*, and *EGFR* (71).

Importantly, tau mutations can be influenced by other genetic and epigenetic factors and may result in heterogeneous clinical syndromes that cannot be well replicated in nonclinical models (72). Because the tau protein sequence is not different between the H1 and H2 haplotypes, pathogenic effects may relate to differences in gene expression or post-transcriptional changes (73). In addition, the association between H1 haplotype and PSP is of somewhat uncertain global significance given the variable haplotype expression in different groups; for example, the H2 haplotype is not present in many Asian populations (74). Further, the H1 haplotype associated with PSP in non-Latinx White populations was not associated with these symptoms in Guadeloupean patients (75).

## Nonclinical (cell culture and animal) tauopathy models

Most evidence in support of tau-targeting therapies is based on experiments in nonclinical models. Historically, tau transgenic mice have been used because they have CNS cell types similar to those of humans and allow for in vivo manipulation of cellular systems. Unfortunately, the predictive value of therapeutic efficacy in mouse models is limited, as large therapeutic effects seen in tau transgenic mouse models have not been replicated in human clinical trials.

Mouse models largely do not develop the neurodegeneration and insoluble tau pathology seen in humans (76, 77). There is no evidence of murine tau fibril formation with age, and very few mouse models accumulate endogenous murine Aβ (78, 79). As a result, transgenic mouse models to study neurodegeneration must express mutant human proteins that lead to rare, severe early-onset disease in humans. A further discrepancy, specifically for AD, is that even the most aggressive mouse models of Aβ accumulation and early plaque development (e.g., the 5xFAD mouse) do not develop secondary murine tau tangle formation or substantial neuronal loss, as seen in human AD (80).

The most common transgenic tau mouse models express familial FTLD–associated (but not AD-associated) *MAPT* mutations (e.g., P301L, P301S) (81–83). These models accumulate hyperphosphorylated tau fibrils and develop phenotypically variable age-dependent synaptic dysfunction, cognitive impairment, and neurodegeneration. Restricting expression of mutated human tau to entorhinal cortex via genetic manipulations results in propagation of tau along connected limbic structures, supporting the “prion-like” hypothesis of tau spread (35). However, there are numerous limitations with these mouse models. First, transgenic mice often express much higher levels of mutated tau throughout the lifespan, which might induce compensatory changes that could either mask pathology or cause phenotypes irrelevant to human disease. Additionally, transgenic tau models only express one of six potential tau isoforms, typically a 4R tau, thus eliminating any potential contribution of alternative splicing or 3R/4R ratios to disease processes. As well, many tau models produce specific tau aggregate strains, which may not be relevant to the human disease in which a particular therapy is eventually tested.

With this knowledge, close attention should be paid to recent tau-directed antibody failures. For example, nonclinical testing of semorinemab was in a mouse model expressing P301L tau, a mutation found in FTLD (usually a behavioral variant FTD phenotype), with subsequent clinical testing in AD patients (84). Conversely, tilavonemab was tested in the P301S model, which expresses an FTLD-only mutation, and then tested in mild-to-moderate PSP and AD patients (4, 85). If tau strain or aggregate structure is key to the development of a particular human disease, failure to accurately target the relevant tau strain could result in a lack of efficacy in human trials.

Other key differences exist between mice and humans that may explain poor translation of mouse tau biology to therapeutics. Murine tau lacks 11 N-terminal amino acids that are present in the human version. These differences in the N-terminus affect tau secretion, protein interaction, and tau phosphorylation (recently reviewed in ref. 86) and may limit the ability of mice to recapitulate nuanced features of disease critical to the development of therapeutics (86). On an organismal level, there are key and relatively unexplored differences in CNS function between mice and humans. For example, microglia may contribute to neurodegenerative disease and show transcriptomic differences between mouse and human, particularly with age (87). Similarly, the blood-brain barrier transcriptome differs between mice and humans, which may impact both disease pathophysiology and the action of peripherally administered drugs (88). In consequence, nonclinical models of tauopathy have at best partially approximated human neurodegeneration — they represent models of possibly relevant disease mechanisms.

Potential alternative models to study tauopathies are in development, including the seeding-based mouse models referenced above. Narasimhan et al. injected pathologic tau from postmortem brains into non-transgenic mouse brains and observed differences in tau strain potency and pathologic localization between AD-tau, CBD-tau, and PSP-tau, such that only PSP-tau and CBD-tau produced glial inclusions, and PSP-tau produced much more extensive tau pathology (89). These models may provide a tool in our arsenal to study the effects of tau treatments on specific aspects of pathology, and tau monoclonal antibodies (mAbs), such as gosuranemab, have been tested in induced pluripotent stem cell (iPSC) cultures seeded with disease-specific tau (90). To address contributions of multiple cell types and aging in vitro, tissue culture methods are becoming increasingly sophisticated. Organoids allow the coculture of multiple human cell types derived from iPSCs, and thus can model the interactions of human microglia, astrocytes, and neurons in vitro (91). Similarly, newer techniques to directly convert patient-derived skin fibroblasts into neurons (iNeurons) bypass the need for an iPSC step and maintain the aging signature of the sample patient skin biopsy (92).

A few rat models of AD also exist that more closely resemble human disease. Specifically, rats expressing mutated human APP develop age-dependent tau pathology and neurodegeneration (93, 94). More research is needed to understand why rats more accurately recapitulate human disease. While the increased cost of housing rats limits their use in many laboratories, rat models may prove a more useful tool for testing therapeutics.

## Therapeutics targeting tau

**Figure 2:**
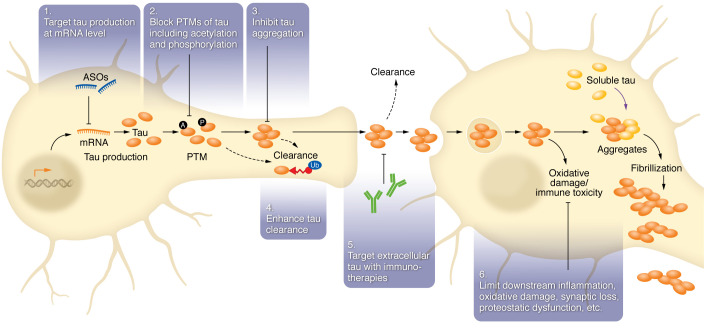
Review of mechanisms for various anti-tau therapeutics. 1. Genetically targeted therapies, such as antisense oligonucleotides (ASOs) and certain small molecules, can target tau production. 2. Small-molecule enzyme inhibitors can target posttranslational modifications (PTMs) such as acetylation (A), phosphorylation (P), and ubiquitination (Ub). 3. Methylene blue derivatives and other aggregation inhibitors were conceived of as targeting tau aggregation. 4. Tau clearance may be enhanced by molecules such as PROTACs (see above). 5. Immunotherapies (vaccines, anti-tau monoclonal antibodies) target extracellular tau. 6. Neuroprotective agents, including antiinflammatory agents, could limit the downstream impacts of tau pathology. Figure adapted with permission from *Neuroscience Letters* (95) and from Martin Kampmann (UCSF) with permission.

Tau therapies have attempted to disrupt toxic gain of function (antisense oligonucleotides/gene therapy), modulate posttranslational modification (PTM), disrupt tau aggregation, passively clear tau, and vaccinate against tau — see Figure 2 for a summary of the classes of therapeutic approaches (95). Conversely, approaches to replace loss of tau physiologic function (microtubule stabilizers) have also been assessed. Though these multiple classes of therapies have been evaluated as disease-modifying agents in human clinical trials (Table 1), therapeutically relevant mechanisms have not been validated.

Notably, the pathogenic tau species has not been definitively identified in living humans. Soluble tau, in the form of oligomers (including dimers), is being explored as a possible source of key neurotoxic species. Alternatively, insoluble tau in the form of both NFTs and other aggregates might represent the toxic species (96). In support of oligomeric soluble tau being important, injection of soluble tau oligomers into wild-type mouse brains, but not injection of tau fibrils or monomers, impaired memory (97).

In general, given the heterogeneity of tau isoforms, tau PTMs, and aggregate structures in tauopathies, some diseases may respond better than others to specific tau-targeting agents. There is cryo–electron microscopic evidence for differences in the structures of tau filaments in different diseases, including Pick’s disease, AD, chronic traumatic encephalopathy (CTE), CBD, globular glial tauopathy (GGT), AGD, and PSP. Particularly, a three-layered fold is noted in PSP and GGT, while a four-layer fold is noted in CBD and AGD (98, 99). Tau seeding models, mentioned above, support the idea that there are differences in tau conformers between pathologies. Experiments involving inoculation of human brain lysates from various tauopathies have revealed brain (neuronal or glial) lesions in mouse models or cell culture that differentially resemble the original human pathology (100). These differences might contribute to differences in efficacy, safety, and tolerability in treatments across tauopathies, as seen in a recent basket trial testing a single intervention in multiple disease groups expressing a common biomarker of a microtubule stabilizer (101).

### Small-molecule PTM inhibitors.

Agents targeting tau PTMs, particularly hyperphosphorylation, have included protein kinase inhibitors that aim to reduce tau aggregation. All of the agents discussed below demonstrated signal in nonclinical models. Concerns with these agents have included potential lack of target specificity and potential for off-target effects. Over 90 phosphorylation sites for tau exist, and specific interventions balancing efficacy with tolerability may be difficult to achieve. Glycogen synthase kinase 3β (GSK-3β) hyperactivity contributes to hyperphosphorylation, which has been considered the major target for pathologic aggregation (102). Lithium inhibits GSK-3β and was evaluated in 17 patients with PSP and corticobasal syndrome; however, it was poorly tolerated due to increased falls, and therefore the trial was stopped (ClinicalTrials.gov NCT00703677). Valproate was also assessed because of anti–GSK-3β activity, but did not improve PSP Rating Scale scores in 28 PSP patients over the course of 2 years (ClinicalTrials.gov NCT00385710) (103). Tideglusib, a novel small-molecule GSK-3β inhibitor, did not demonstrate evidence of efficacy in mild to moderate AD (ARGO, NCT01350362) or in PSP (TAUROS, NCT01049399) (104, 105). Another kinase implicated in tau hyperphosphorylation, Fyn, has been targeted by a small-molecule inhibitor (saracatinib) in a phase II trial of patients with mild AD (CONNECT, NCT02167256), which was stopped for lack of clinical efficacy and concern for gastrointestinal side effects (106).

O-GlcNAcylation (OGA) targeting may decrease hyperphosphorylation, and a small-molecule inhibitor (MK-8719) showed nonclinical mouse model signal in decreasing tau aggregation, but did not advance to phase II clinical trials in humans (107). Other OGA-targeting agents also await evaluation in phase II studies; however, LY3372689 is currently in a phase II AD trial (NCT05063539).

Tau acetylation can prevent physiologic clearance; salsalate, a small-molecule acetylation inhibitor, did not show a treatment effect in a futility study of 10 patients with PSP, nor was there evidence of efficacy in a small randomized, placebo-controlled trial in mild to moderate AD (presented in abstract form at the Clinical Trials on Alzheimer’s Disease conference in 2022) (108, 109).

Tau aggregation disruption aims to prevent the paired helical filament conformation observed in NFTs, and a derivative of methylene blue (LMTM), which prevents this in mouse models, was evaluated in a phase III trial of behavioral variant FTD without evidence of efficacy (110). Multiple phase III trials in AD, most recently LUCIDITY (NCT03446001), have also been negative based on prespecified analyses.

Microtubule stabilization designed to ameliorate putative loss of physiologic function has been attempted. Davunetide is derived from activity-dependent neurotrophic protein (ADNP), a neuroprotective agent that decreased hyperphosphorylated tau in nonclinical models through an unclear mechanism. It did not demonstrate any clear benefits in randomized trials in 144 patients with mild cognitive impairment nor in 313 patients with PSP (111, 112). Abeotaxane (TPI-287), a microtubule stabilizer, produced anaphylactoid reactions in patients with AD but not PSP in a basket-design clinical trial with patients with AD and 4R tauopathies; it also led to a dose-related worsening of function and more frequent falls in 4R tauopathies (101).

Antisense oligonucleotides (ASOs) are directed against *MAPT* mRNA to reduce tau expression. This strategy is based on data in mouse models showing that reducing human tau expression improves hippocampal volume loss and cognitive deficits (113). In this same work, CNS penetration was demonstrated in primate models. Results are pending in a study of tau lowering with ASO (BIIB080) in 64 patients with mild AD, but preliminarily ASO therapy reduced tau levels in cerebrospinal fluid (CSF), reduced MK-6240 tau PET uptake, and was well tolerated (114). A phase II trial in AD is now enrolling (NCT05399888), and a similar phase I trial with a different tau ASO is ongoing in PSP (NCT04539041).

Improvement of tau clearance has been assayed using proteolysis-targeting chimera (PROTAC) molecules to selectively enhance ubiquitination and proteolysis of tau proteins, as demonstrated in nonclinical models, including patient-derived neural cell models (115, 116). There are no currently running human clinical trials.

### Immune therapies.

Both active (vaccine) and passive (mAb-mediated) immune therapies are being investigated in tauopathies.

AADvac1 was the first tau-directed vaccine tested in trials, employing a truncated version of the tau protein that was thought to be the pathogenic fragment in the MTBR triggering aggregation. Immunogenicity was demonstrated in a phase I trial, but unfortunately in a phase II trial versus placebo in mild AD dementia, slowing of cognitive and functional decline was not demonstrated, although it slowed the increase in blood neurofilament light chain (117). Another trial of a liposome-based vaccine (ACI-35) targeted toward pathologic phosphorylation residues is under way (118).

Monoclonal antibodies targeting the N-terminal tau domain have been tested in multiple phase II trials, largely without clinical benefit despite evidence for target engagement via reduction of N-terminal CSF tau. These trials have included gosuranemab (in PSP and early AD), tilavonemab (in PSP and AD), and zagotenemab (119). Notably, in a trial of mild-to-moderate AD (in contrast to prodromal to mild AD), semorinemab, also an N-terminal IgG4 antibody, led to a 43.6% slowing of decline on the Alzheimer’s Disease Assessment Scale–Cognitive Subscale (ADAS-Cog) co–primary outcome measure, in the absence of benefit for the other cognitive or functional outcomes. Whether this was due to chance or a true therapeutic effect is a topic of debate (phase II LAURIET trial, NCT03828747). If true, it is unclear why a therapeutic effect was absent in an earlier phase of AD, but it could be hypothesized that different species of tau more amenable to semorinemab engagement predominate in later stages of the disease; there may be higher concentrations of N-terminal tau fragments in later-stage disease if they are related to the overall amount of cortical tau pathology (soluble or insoluble). In addition, the mid-region, MTBR, and C-terminal tau–targeting antibodies bepranemab, E2814, LuAF87908, and JNJ-63733657 are in phase I–II trials and may have better clinical effect given the importance of the MTBR and C-terminus in tau aggregate structure.

Another possible reason for the lack of observed clinical benefit in trials is that mAbs have targeted extracellular tau. This mechanism was thought to be valuable on the basis that extracellular tau may undergo spread to other neurons (as demonstrated in nonclinical models). However, it is unknown whether recent tau mAbs have reached a high enough concentration in the brain parenchyma to affect these species, since there are no human biomarkers to measure soluble tau levels in the brain parenchyma. By analogy to anti-amyloid antibodies, it may be necessary to activate immune-mediated clearance for efficacy, but most anti-tau mAbs tested have been IgG4 with reduced effector domain, which is the least effective isotype to promote microglia phagocytosis (120). Further, to bypass systemic circulation and ensure cerebral delivery at correct levels, adeno-associated viral antibody delivery may be an avenue (121). Interestingly, recent work demonstrated that tau immunotherapy may rely on the intracellular antibody receptor TRIM21 (122). Mice lacking expression of TRIM21 were nonresponsive to tau-targeting immunotherapy both at an early stage of tau pathogenesis and during prolonged treatment, which may have implications for tau mAb treatment in human disease. Optimization of antibody characteristics, including isotype, epitope, charge, affinity, size, vehicle, and timing of delivery, may also be important for identifying an efficacious approach (120).

It is also important to consider patient effects, including aging, on changes in effectiveness of immune therapies such as vaccination and antibody therapies, related to alterations in the B and T cell compartments termed immunosenescence (123). Decreased tau clearance related to aging, such as through the glymphatic system, may also be therapeutically relevant; even if tau is targeted appropriately by therapies, it may still not be cleared (124). These phenomena should be accounted for in designing such therapies in tauopathies, perhaps with dose and schedule differences (125).

## Tau biomarkers

In recent years, multiple in vivo biomarkers for tau pathology have been evaluated; these are key to detection of tau pathology, clinical trial enrollment, and assessment of the efficacy of tau therapeutics. These are overall better validated in AD than in other tauopathies. Currently, no biomarkers are approved for diagnosing non-AD tauopathies or for following the clinical course of any tauopathies.

The first tau PET tracer, [^18^F]flortaucipir, was approved for clinical use for the detection of AD by the US Food and Drug Administration (FDA) in May 2020 (126). This tracer was less sensitive to tau related to FTLD spectrum disorders (127). Newer tau PET tracers, including [^18^F]PI2620, likely bind more selectively to hippocampal tau related to AD but may also have utility for identifying 4R tauopathies (128, 129).

Fluid biomarkers for tau pathology include serum and CSF tau assays. Plasma p-tau181, p-tau217, and p-tau231 are promising and potentially more easily accessible biomarkers (130). In particular, plasma p-tau217 has shown utility in combination with tau PET for staging AD pathology (131, 132). Elevated CSF total tau and p-tau (most commonly p-tau181) are also suggestive of an AD pathology (133). Combining different markers, including Aβ and neurofilament light chain, can yield better discriminability of CSF tau for FTLD spectrum disorders (134). CBD may be distinguished from other tauopathies by incorporation of differences in specific CSF MTBR tau fragments, a finding that should be further explored (135).

Distinguishing AD from other tauopathies or identifying when they co-occur is important, as co-occurrence is common and may have therapeutic implications. One goal for future research is to design tau biomarkers with increased sensitivity and specificity for the early differential diagnosis of tauopathies and their longitudinal progression.

## Next steps in designing tau-targeting therapies

In this Review, we have outlined multiple potential reasons for the lack of success to date in the tau-targeting therapies that have come to human clinical trials. These include poorly predictive nonclinical models, an inability to relate specific models to specific human diseases, targeting of the wrong tau species (N-terminal tau) or pathogenic mechanism (phosphorylation), difficulty in designing the optimal immunologic approach, lack of biomarkers to diagnose early-stage tauopathies and to measure treatment response, the possibility that recent trials have started too late in the course of disease, and insufficient numbers of clinical trials in different human tauopathies that could respond differently to the same tau therapy.

It is clear that a novel approach to identifying and testing therapies in humans is needed. We know that nonclinical models are imperfect and that some phenomena studied in these models may not be therapeutically relevant to humans. Further, secondary effects of tau pathology, such as aggregation of other pathogenic proteins and neuroinflammation, may not be addressed by therapies that solely target tau. Timing is also critical: it is possible that even mild cognitive impairment is too late with regard to the development of pathology leading to neurodegeneration in humans. Or perhaps, as suggested by semorinemab’s failure to slow progression of early and mild AD, it is too early?

There is an urgent need to bring therapies to the clinic setting for all patients with neurodegenerative disease, including tauopathies. We believe it is time to refocus on “interventional” human research, as a departure from the current focus on therapeutic design, which entails years of expensive work on nonclinical models. Once therapeutic safety is established in early-phase trials, new approaches will be necessary to efficiently and effectively evaluate multiple therapeutic mechanisms in parallel. Training and departmental support for academic clinical trialists to carry out this work should be prioritized. Therapeutic classes and agents targeting different mechanisms can be tested in basket trials to enhance drug development efficiency by evaluating the effects of one therapy in multiple tauopathies (136). Umbrella trials of multiple agents in one disease also have utility in tauopathies, as exemplified by the current combination trial of anti-amyloid (lecanemab) and anti-tau (E2814) treatments in dominantly inherited AD (DIAN-TU) (137). Pragmatic trials, which assess effectiveness in the real-world clinic setting, of existing, repurposable drugs, such as symptom-targeting medications, have been successfully conducted in other neurologic conditions and might also be prioritized (138). Disease progression models of existing data have been applied in rare familial FTD (*MAPT* mutation carriers) to leverage surrogate biomarker endpoints (neurofilament light chain and MRI) to select the optimal inclusion criteria and endpoints to maximize power to detect treatment effects (139).

There are gaps in our understanding of the pathobiology of tauopathies, but regardless, an overwhelming amount of circumstantial evidence implicates tau protein as a driver of human disease, particularly in the primary tauopathies. As the science of tau therapy and clinical trials advances, there are likely to be important and unexpected insights into the pathogenic mechanisms of tauopathies that will identify novel agents that should be efficiently tested in clinical trials. Overall, we are optimistic about the future of tau-targeted therapies and our ability as a field to bring them to patients, as we continue to refine our understanding of tau biology and drug development.

## Figures and Tables

**Table 1:**
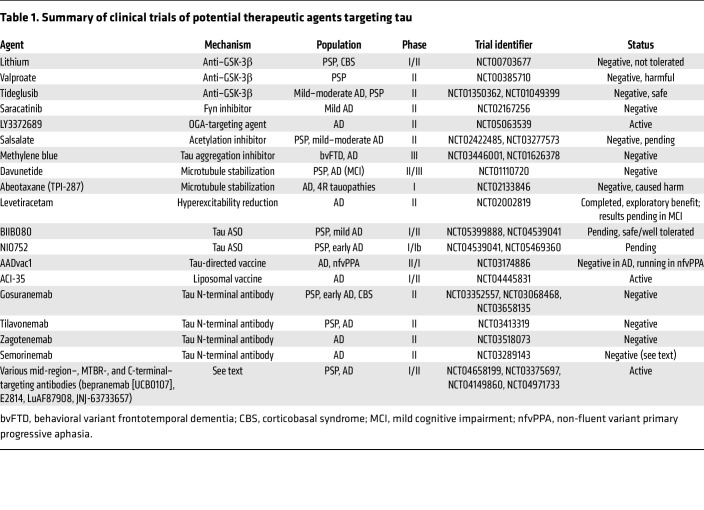
Summary of clinical trials of potential therapeutic agents targeting tau

## Version 1 06/15/2023

Electronic publication
